# Spatial Heterogeneity and Its Influencing Factors of Syphilis in Ningxia, Northwest China, from 2004 to 2017: A Spatial Analysis

**DOI:** 10.3390/ijerph191710541

**Published:** 2022-08-24

**Authors:** Ruonan Wang, Xiaolong Li, Zengyun Hu, Wenjun Jing, Yu Zhao

**Affiliations:** 1 Department of Epidemiology and Health Statistics, School of Public Health and Management, Ningxia Medical University, Yinchuan 750001, China; 2 Ningxia Key Laboratory of Environmental Factors and Chronic Disease Control, 1160 Shengli Street, Xingqing District, Yinchuan 750001, China; 3 State Key Laboratory of Desert and Oasis Ecology, Xinjiang Institute of Ecology and Geography, Chinese Academy of Sciences, Urumqi 830011, China; 4 School of Statistics, Shanxi University of Finance and Economics, Taiyuan 030006, China

**Keywords:** syphilis, SMR, *Moran* *’s I*, spatial cluster, spatial regression model

## Abstract

Syphilis remains a growing and resurging infectious disease in China. However, exploring the influence of environmental factors on the spatiotemporal distribution of syphilis remains under explore. This study aims to analyze the spatiotemporal distribution characteristics of syphilis in Ningxia, Northwest China, and its potential environmental influencing factors. Based on the standardized incidence ratio of syphilis for 22 administrative areas in Ningxia from 2004 to 2017, spatiotemporal autocorrelation and scan analyses were employed to analyze the spatial and temporal distribution characteristics of syphilis incidence, while a fixed-effect spatial panel regression model identified the potential factors affecting syphilis incidence. Syphilis incidence increased from 3.78/100,000 in 2004 to 54.69/100,000 in 2017 with significant spatial clustering in 2007 and 2009–2013. The “high–high” and “low–low” clusters were mainly distributed in northern and southern Ningxia, respectively. The spatial error panel model demonstrated that the syphilis incidence may be positively correlated with the per capita GDP and tertiary industry GDP and negatively correlated with the number of health facilities and healthcare personnel. Sex ratio and meteorological factors were not significantly associated with syphilis incidence. These results show that the syphilis incidence in Ningxia is still increasing and has significant spatial distribution differences and clustering. Socio-economic and health-resource factors could affect the incidence; therefore, strengthening syphilis surveillance of migrants in the economically developed region and allocating health resources to economically underdeveloped areas may effectively help prevent and control syphilis outbreaks in high-risk cluster areas of Ningxia.

## 1. Introduction

Syphilis is a chronic infectious disease, commonly caused in humans by *Treponema pallidum* (TP) infection. It principally spreads through sexual contact, vertical transmission, and blood transmission [1,2]. Early signs of syphilis usually manifest as genital and skin mucous membrane damage, while late syphilis can involve the bones, nerves, and cardiovascular system. [3,4]. According to the 2021 WHO Global Progress Report on HIV, Viral Hepatitis and Sexually Transmitted Diseases, an estimated 7.1 million new cases of syphilis (95% UI: 3.8–10.3 million) were reported worldwide in 2020, an increase of nearly 800,000 from over 6.3 million in 2016 [5,6]. As one of the class B notifiable infectious diseases in China, the reported incidence of syphilis in 2020 was the third highest after viral hepatitis and tuberculosis [7]. Thus, syphilis is still one of the most severe sexually transmitted infection, warranting global prevention and control since it can cause serious harm to human health.

About 80% of epidemiological data have spatial attributes [8]; thus, analyzing the spatiotemporal distribution characteristics of syphilis incidence and further exploring the meteorological or socio-economic driver factors associated with the spatiotemporal heterogeneity of syphilis incidence can promote the development of effective responses for high-risk areas [9,10,11,12]. Liu et al. [13] suggests that the rapid spread of syphilis may be the result of a combination of biological and social factors. From the perspective of geographical distribution of syphilis incidence, the affected regions in China gradually expanded from southeast coastal areas with relatively rapid economic development regions to north and southwestern inland areas [14,15,16]. From the perspective of associated meteorological or socio-economic factors, previous studies have shown inconsistent results concerning associated factors of syphilis incidence. For example, Smock et al. [17] found that syphilis incidence was higher in poverty-level groupings. However, Read et al. [18] showed that economically developed areas had a higher risk of syphilis. Thus, syphilis incidence in different regions showed a significant spatial discrepancy and conflicting results regarding the potential associated socio-economic or meteorological factors.

Identifying key areas of syphilis incidence based on spatiotemporal distribution studies, and combining evidence of potential meteorological or socio-economic drivers to adjust syphilis prevention strategies and allocate health resources is of great significance for effective syphilis prevention and control. Current spatial econometric methods are widely used in public health and health policy evaluation [19,20,21,22]. Tang et al. [11] applied a spatial panel data model to explore the relationship between socio-demographic factors, socio-economic factors, and the incidence of primary and secondary syphilis in Guangdong China after controlling for spatial effects. Salway et al. [23] described the spatial-temporal epidemiology of infectious syphilis and identified the associations between neighborhood-level factors and rates of syphilis in British Columbia, Canada. Understanding the potential drivers of the incidence of syphilis has developed from traditional epidemiology to spatial epidemiology; however, there are still few related studies on its potential influencing factors.

Ningxia Hui Autonomous Region (referred as “Ningxia”), located in northwest China, is an economically underdeveloped region, bordering the Gansu and Shaanxi provinces. According to a report by the Ningxia Center for Disease Control and Prevention, syphilis remains a growing and resurging infectious disease. Existing evidence showed that socio-economic and health-resource factors may affect the spatial distribution of syphilis [11]; however, there are very few result related to the association between these factors and spatial heterogeneity of syphilis in Ningxia. To fill this gap, we used spatial autocorrelation and scan analyses to investigate the spatiotemporal distribution characteristics of syphilis in Ningxia, based on the number of reported syphilis cases in 22 administrative areas in the Ningxia region from 2004 to 2017. We then constructed a spatial panel regression model to explore the potential socio-economic, health-resource factors, and meteorological factors that influence the distribution of syphilis incidence. These results may provide a theoretical basis for precisely adjusting syphilis prevention and control in high-risk regions of Ningxia.

## 2. Materials and Methods

### 2.1. Study Area

In this study, 22 administrative areas across five cities in Ningxia, China were selected as the study area. The specific research areas were as follows:

(i) Yinchuan city: including Xingqing, Xixia, Jinfeng, Yongning, Helan, and Lingwu;
(ii) Shizuishan city: including Dawukou, Huinong, and Pingluo;
(iii) Wuzhong city: including Litong, Hongsibu, Qingtongxia, Yanchi, and Tongxin;
(iv) Guyuan city: including Yuanzhou, Xiji, Longde, Jingyuan, and Pengyang;
(v) Zhongwei city: including Shapotou, Zhongning, and Haiyuan.

### 2.2. Data Sources

#### 2.2.1. Syphilis Incidence Data

Data on the syphilis incidence in the Ningxia Hui Autonomous Region from 2004 to 2017 were obtained from the National Notifiable Infectious Disease Reporting Information System (as shown in Figure S1). All the case data were de-identified, and only the onset year and region of the case were retained.

#### 2.2.2. Geographic Information Data

The Ningxia map base layer comes from the National Basic Geographic Information System (http://www.ngcc.cn/ngcc/ (accessed on 22 January 2022)).

#### 2.2.3. Meteorological Factors, Socio-Economic Data, and Health-Resource Data

In addition, the administrative area-level meteorological factors, socio-economic, and health-resource data of 22 administrative areas in Ningxia from 2004 to 2017 were obtained from Ningxia Statistical Yearbook (http://nxdata.com.cn/publish.htm?cn=G01 (accessed on 22 January 2022)), as shown in Table S1.

### 2.3. Statistical Analysis

#### 2.3.1. Descriptive Analysis

The annual population composition and syphilis incidence rate of each administrative area in Ningxia were used as criteria to calculate the standardized morbidity ratio (SMR) of syphilis [24], the formula used is as follows:

(1) $$SMR=\frac{y_{ij}}{E_{ij}},$$

Here, *y_ij_* denotes the number of reported syphilis cases in administrative area *i* (1 ≤ i ≤ 22) in year *j* (2004 ≤ *j* ≤ 2017) and *E_ij_* denotes the expected number of reported cases in the administrative area *i* in year *j*, which can be obtained by multiplying the population of the administrative area by the reported incidence of syphilis in the whole district in year *j*.

#### 2.3.2. Three-Dimensional Trend Surface Analysis

To visualize the spatiotemporal variations of the incidence of syphilis in Ningxia from 2004 to 2017, the three-dimensional spatial trend surface of the average incidence of syphilis in different areas was analyzed to reveal the spatial distribution characteristics of the clusters [25]. Each disease index value is regarded as a point value located at the geometric center, and is scattered in a three-dimensional space with the disease index as the Z-axis, and the longitude and latitude of different geographic locations as the X-axis and Y-axis, respectively. In the figure, the scatter points of the disease index data are projected to the XZ plane and the YZ plane, respectively, and the data is fitted. The estimated value reflects the change and development trend of the research objects in the entire region. The line on the XZ plane corresponding to the Y axis represents the trend change of the disease index in the north-south direction (the latitude), and the arrow points to the north; the line on the YZ plane corresponding to the X axis represents the east-west direction (i.e., the longitude), and the arrow points to the east.

#### 2.3.3. Spatial Autocorrelation Analysis

The global *Moran’s I* and the local *Moran’s I* are used as indicators of spatial autocorrelation analysis. The range of *Moran’s I* is from −1 to 1; I > 0 indicates positive spatial correlation. The closer the value is to 1, the higher the spatial aggregation is. I < 0 indicates negative spatial correlation; I = 0 indicates that there is no spatial aggregation, that is, random distribution [26,27]. Local *Moran’s I* mainly includes four clusters: high–high cluster (high–high), low–low cluster (low–low), high–low cluster (high–low), and low–high cluster (low–high) [28,29].

#### 2.3.4. Spatiotemporal Cluster Analysis

In order to further identify the spatiotemporal clustering characteristics of high-risk of syphilis in Ningxia and determine the location and relative risk of the clusters, the spatial scan analysis performed by SaTScan was employed [30,31]. The maximum scanning window was set as 50% of the total population and 50% of the research period in this study [32,33]; different clustering regions were detected by the logarithmic Likelihood Ratio (abbreviated as *LLR*). The size and position of the risk area circles are drawn according to the result file of ArcGIS version exported from the spatiotemporal scan analysis (SaTScan 9.4.1, Martin Kulldorff, Boston, MA, USA).

#### 2.3.5. Spatial Panel Data Model

Spatial panel data models were employed to identify the potential meteorological influencing factors of syphilis. Spatial regression models included: Spatial Lag Model (SLM/SAR), Spatial Error Model (SEM), and Spatial Durbin Model (SDM). Lagrange multiplier test (LM test) and Hausman test (HAUSMAN test) were required to determine which spatial regression model would be most appropriate [20,22,34]. As illustrated in Figure S1, the model creation steps are roughly as follows:

(1) Lagrange multiplier test (LM test): It is used for the selection of mixed ordinary least squares (OLS) regression model and spatial regression model. If *p* < 0.05, then the mixed OLS regression model should be rejected and the spatial panel regression model should be used.
(2) Robustness test: Likelihood ratio test (LR test) and Wald test are used to test whether the preset SDM model can degenerate into SLM or SEM model. When the result is significant (*p* < 0.05), it cannot be degraded to SLM or SEM model, and SDM model should be used.
(3) Model effect selection:
  (i) Selection of random/fixed effects: The Hausman test (HAUSMAN test) is used. The coefficients obtained from this test are generated under random effects. If *p* < 0.05, H_0_ is rejected and fixed effects should be selected.
  (ii) Selection of individual/time/two-way effect: The individual and time effects are compared with the two-way effect. If the test results are both significant (*p* < 0.05), the two-way effect will be used.
(4) Based on the above test processes, a reasonable spatial regression model is established.

The spatial error model with spatial and temporal fixed effects could be specified as follows:

(2) $$z_{it}=\mu_{i}+\gamma_{t}+X_{ij}\beta +\phi_{it},\phi_{it}=\lambda \sum_{j=i}^{N}W_{ij}\phi_{ij}+\varepsilon_{it},\;\phi_{it}=\lambda \sum_{j=i}^{N}W_{ij}\phi_{ij}+\varepsilon_{it},$$

where $z_{it}\;$ denotes the SMR of syphilis in administrative area *i* (1 ≤ *i* ≤ 22) at year *t*; $\mu_{i}$ and $\gamma_{t}\;$ are the spatial specific effect and the temporal specific effect, respectively. $X_{ij}$ is a set of independent variables in administrative area *i* at year t, β is the regression coefficient and $\phi_{it}$ is the spatial related random error in administrative area i at year t. $W_{ij}$ is an N×N positive non-stochastic spatial weight matrix to represent the spatial association of counties, λ is the coefficient of spatial autoregression, $\varepsilon_{it}$ is the random error.

### 2.4. Statistical Software

Statistical description and spatial regression models were generated by Stata 14.0, three-dimensional spatial trend surface analysis was completed by ArcGIS10.6, spatiotemporal scan analysis was performed by SaTScan 9.4.1 (Martin Kulldorff, Boston, MA, USA), and spatial autocorrelation analysis was performed by GeoDa 1.10 (Arizona State University, Phoenix, AZ, USA) and ArcGIS10.6, with the number of Monte Carlo randomized repeated simulations set as M = 999. The significance level of *p* < 0.05 was regarded as statistically significant.

## 3. Results

### 3.1. Description of Spatiotemporal Distribution

**Figure 1:**
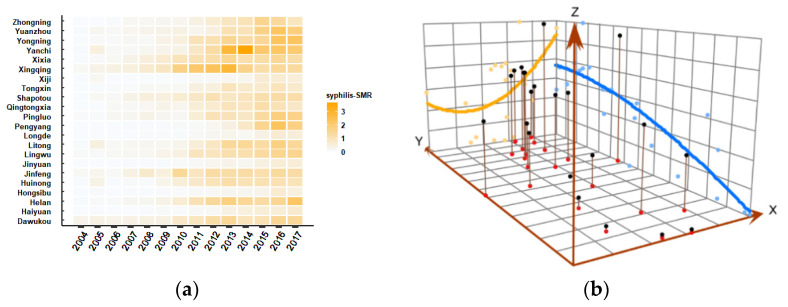
Syphilis in 22 administrative areas of Ningxia, China. (**a**) Heat map of SMR of syphilis in 22 administrative areas of Ningxia, (**b**) spatial three-dimensional trend surface analysis of the mean SMR of syphilis in Ningxia (SMR: standardized morbidity ratio). The blue and yellow lines are the trend fitting lines of syphilis SMR in the north–south and east–west directions, respectively.

A total of 28,621 syphilis cases were reported in Ningxia from 2004 to 2017. Spatiotemporal analysis of syphilis SMR in Ningxia from 2004 to 2017, as shown in Figure S2, illustrated that the incidence of syphilis in Ningxia increased from 3.78/100,000 in 2004 to 54.69/100,000 in 2017, and syphilis in Ningxia has significant spatial heterogeneity. More precisely, the administrative areas with higher syphilis SMR were mainly located in northern areas of Ningxia, such as Dawukou, Xingqing, Jinfeng, Yanchi, Litong, etc. (Figure 1a and Table S2). The darker the color, the larger value of syphilis SMR.

The results of spatial three-dimensional trend map revealed that the distribution of syphilis SMR in the 22 administrative areas in Ningxia was higher in the northern regions than in the southern regions in the north-south direction, and the distribution in the eastern regions was higher than the western region in the east-west direction. The results of trend surface analysis indicated that syphilis was a more serious problem in the eastern and northern regions of Ningxia (Figure 1b).

### 3.2. Spatiotemporal Scan Analysis

**Figure 2:**
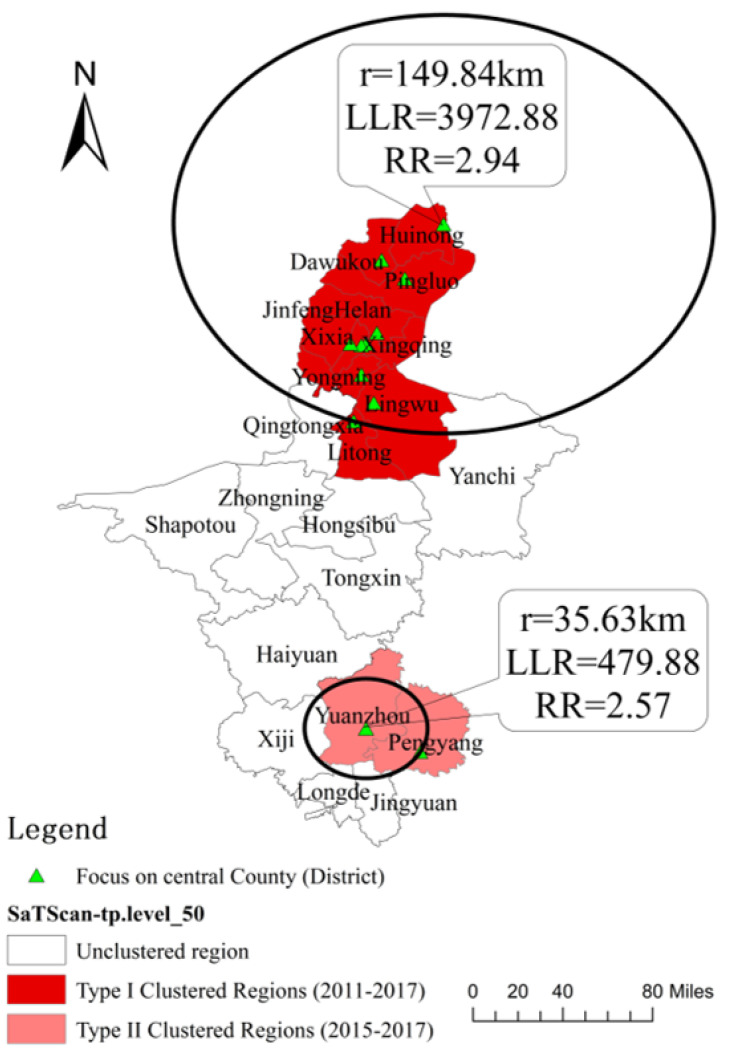
Spatial and temporal scan distribution of syphilis incidence in Ningxia, 2004–2017. Note: r is the radio of high-risk spatiotemporal clustering, *LLR* is the log likelihood ratio and *RR* is the relative risk of clustering area.

**Figure 3:**
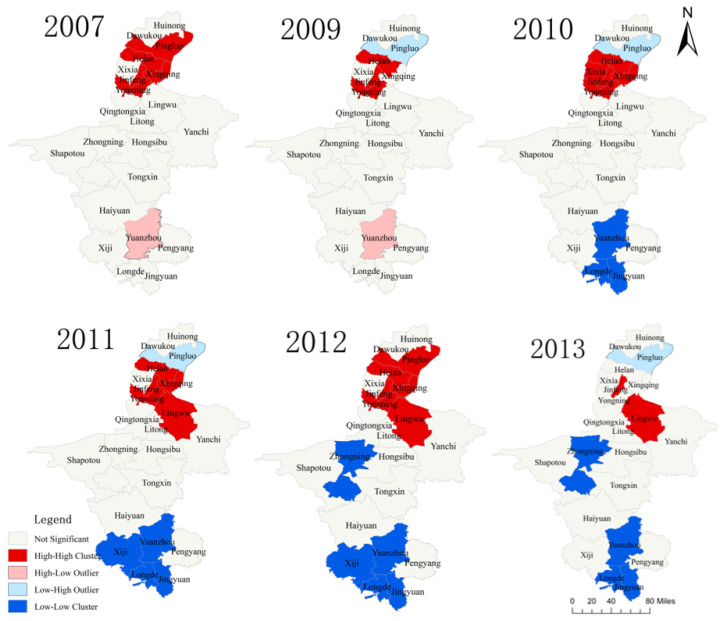
Local indices of spatial association aggregation plots of syphilis SMR values in Ningxia.

As shown in Table 1 and Figure 2, the maximum scan window was set to 50% of the total population and 50% of the study period and two spatiotemporal clusters were detected in Ningxia. Considering the spillover effects across adjacent regions, the circle in Figure 3 indicated the high-risk spatiotemporal clustering area of syphilis in Ningxia, which is an effective measure to quantitatively identify the high risk areas. The first cluster occurred from 2011 to 2017 in north Ningxia, which covered 10 administrative areas, such as, Huinong, Dawukou, Pingluo, Helan, Xingqing, Jinfeng, Xixia, Yongning, Lingwu, and Litong, with a radius of 149.84 km. The risk of syphilis in this cluster was 2.94 times higher than that found in the other areas (*LLR* = 3972.88, *p* < 0.001). The second cluster occurred in south Ningxia during 2015–2017, covering Yuanzhou and Pengyang, with an area radius of 35.63 km, and the risk of syphilis in this cluster was 2.57 times higher than that found in the other areas (*LLR* = 594.84, *p* < 0.001).

### 3.3. Spatial Autocorrelation Analysis of Syphilis SMR

The *Moran’s I* index was used to analyze the spatial autocorrelation of syphilis incidence in 22 administrative areas of Ningxia from 2004 to 2017. As illustrated in Table 2, the results demonstrated that the SMR of syphilis in Ningxia had a significant positive spatial correlation in 2007, 2009, 2010, 2011, 2012, and 2013, respectively (*Moran’s I* > 0, *p* < 0.05). The spatial cluster of syphilis in Ningxia was the highest in 2012 (*Moran’s I* = 0.5439) and lowest in 2007 (*Moran’s I* = 0.2369).

To further explore the spatiotemporal clusters of syphilis in Ningxia, the local *Moran’s I* was used. We drew the scatter diagram of *Moran’s I* and LIAS aggregation diagram. The results are shown in Table S3 and Figure 3. The SMR of syphilis in Ningxia shows a positive spatial correlation with spatial clusters. The spatial autocorrelation of syphilis in 2007, 2009, 2010, and 2013 is lower than that in other years. The “high–high” cluster areas of syphilis are mainly in the northern regions of Ningxia, such as Pingluo, Helan, Jinfeng, Xingqing, and Yongning, while the “low–low” clustering areas of syphilis are mainly in the southern regions of Ningxia, e.g., Zhongning, Yuanzhou, Xiji, Longde, and Jingyuan.

### 3.4. Spatial Regression Models

Syphilis in Ningxia showed a significant spatial discrepancy. In order to further explore the potential factors associated with the spatiotemporal distribution differences, we established the spatial regression model to identify the factors influencing meteorological, socio-economic, and health-resource factors. Table 3 provides the descriptive statistics of meteorological, socio-economic, and health-resource factors in Ningxia.

It follows from the LM test, that the serial autocorrelation and spatial error effect should be considered (Table 4). According to the HAUSMAN test in Table 5, fixed effect model should be selected (*χ*^2^ = 24.25; *p* = 0.0117). Thus, the fixed-effect spatial error model was more suitable to adopted.

The fixed-effect spatial error model was constructed to consider the time fixed effect, individual fixed effect, and bidirectional fixed effect (see Table 6 for more details). As shown in Table 7, the comparison of individual fixed effect, time fixed effect, and bidirectional fixed effect showed that the statistical results were all statistically significant (*p* < 0.05). Therefore, it is more reasonable to choose the spatiotemporal bidirectional fixed effect model to investigate the potential influencing factors.

Table 8 illustrated that there was a positive correlation between SMR of syphilis and GDP per capita (*Z* = 2.180, *p* = 0.029) and tertiary industry GDP (*Z* = 1.800, *p* = 0.072) in Ningxia, indicating that higher GDP per capita and tertiary industry GDP adversely affect the incidence of syphilis. Moreover, the SMR of syphilis in Ningxia was negatively correlated with the number of healthcare facilities (*Z* = −1.670, *p* = 0.094) and the number of healthcare personnel (*Z* = −1.98, *p* = 0.047), which implied that the adequate medical resources was conducive to the prevention and control of syphilis. Moreover, the coefficient of spatial autoregression was λ = 0.355 (*p* < 0.001), which demonstrated that the aforementioned influencing factors, were also affected by the neighborhood levels.

## 4. Discussion

In order to effectively strengthen syphilis prevention and control, the 59th World Health Assembly adopted the Prevention and Control of Sexually Transmitted Infections: Draft Global Strategy [35] in 2006, and in 2010 China formulated the China Syphilis Prevention and Control Plan (2010–2020) [36] based on its national situation and relevant regulations, aiming to reduce the incidence of primary and secondary syphilis by 2020. However, the goal of controlling syphilis infection in China has yet to be achieved. Early prevention of syphilis can effectively stop its transmission. By identifying the spatial high-risk regions of syphilis incidence and its relationship with the socio-economic, health resource, and meteorological influencing factors in different regions may provide a deeper insight into early prevention and control strategies of syphilis. Based on the yearly incidence of syphilis during 2004–2017 in Ningxia, this study explored the spatiotemporal distribution of syphilis and investigated the association between the incidence of syphilis and meteorological factors, socio-economic, and health-resource. We found that the overall reported incidence of syphilis in Ningxia presented a yearly increasing trend, rising from 3.78/100,000 in 2004 to 54.69/100,000 in 2017, and this epidemiological trend was generally consistent with the findings of other regions in China [14,37,38] and higher than the national incidence level (34.49/100,000) in 2017 [7].

Spatial autocorrelation methods were used to analyze the spatiotemporal distribution characteristics of syphilis incidence in Ningxia from 2004 to 2017. There were clusters of syphilis incidence in Ningxia in 2007, 2009, 2010, 2011, 2012, and 2013. The “high–high” cluster areas were mainly in northern Ningxia (Helan, Jinfeng, and Pingluo), surrounding Yinchuan, the provincial capital of Ningxia. The “low–low” clusters were mainly located in southern Ningxia, with a tendency of distribution towards the central regions. This result was consistent with the findings of the spatiotemporal scan analysis, which confirmed that two spatial-temporal clusters were detected in Ningxia, the first cluster was identified during 2011–2017 in north of Ningxia (area radius of 149.84 km) and the second cluster was identified in south Ningxia during 2015–2017 (area radius of 35.63 km) with 2.94- and 2.57-times higher risk of syphilis than that found in other areas, respectively. The results of the three-dimensional trend surface analysis demonstrated that syphilis was more prevalent in the eastern and northern regions of Ningxia. It may be related to the continuous socio-economic development and change in people’s ideology in recent years; the gradual opening up of attitudes toward sexual behavior and presence of risky sexual behaviors results in a significant increase in the chance of infection contraction, which may explain the rapid increase in the number of syphilis cases [14,38]. Therefore, it is necessary to strengthen the early screening of syphilis in high-risk areas and conduct health promotion activities for high-risk population, which promote the use of condoms to minimize contact infection, establish correct sexual morality, and improve health awareness [39,40].

Regarding the factors influencing the spatiotemporal distribution of syphilis incidence, this study included meteorological, socio-economic, and health-resource indicators into the spatial error model for exploratory analysis. After adjusting for the effects of spatial autocorrelation, the results showed that the incidence of syphilis in Ningxia may be related to the tertiary industry GDP, per capita GDP, and the number of healthcare personnel. The incidence of syphilis in Ningxia may be positively correlated with the tertiary industry GDP and per capita GDP, while negatively correlated with the number of healthcare facilities and healthcare personnel. However, sex ratio and meteorological factors were not significantly associated with syphilis incidence in Ningxia.

Increasing 1 unit of tertiary industry GDP and GDP per capita may lead to an increase of 3.19 × 10^−7^ and 4.38 × 10^−6^ units of syphilis SMR, respectively. The socio-economic factors may play an important role in aggravating the high-risk spatial clustering of syphilis at the neighborhood level. With the rapid development of tertiary industry and the acceleration of urbanization, an increasing migrant population may be partially responsible for the high-risk regions located in the economically developed areas of north Ningxia. The research of related scholars [41,42] shows that, compared with other urban agglomerations in the northwest region, the inter-city flow in Ningxia along the Yellow City agglomeration is more obvious; the population mobility in the economically developed areas of Ningxia is increasing year by year, such as Yinchuan had a floating population of 418,500 at the end of 2018. In addition, Wu et al. [43] found that the presence of migrant laborers was associated with primary/secondary syphilis infection in Shenzhen. Several empirical studies have also shown that subsets of rural-to-urban migrants have increased sexual risk [21,44,45]. Thus, strengthening syphilis surveillance of migrant populations in the high-risk, economically developed, northern region of Ningxia may be an effective measure to effectively prevent syphilis. In addition, the spatial error model and spatial scan analysis also suggested that more prevention and control measures were needed to be disseminated to the neighborhoods of high-risk clusters.

Our results provided an insight into the relationship between allocation of health resources and incidence of syphilis in Ningxia. The incidence of syphilis was negatively correlated with the number of healthcare personnel. This may be related to the imbalance in the construction of medical institutions, recruitment of healthcare personnel, and the inadequate capacity for diagnosis and treatment of sexually transmitted diseases in medical institutions in each administrative area. It is suggested that rational allocation of medical resources can reduce the incidence of syphilis to a certain extent. Consistent with previous studies [11,39,46,47], developed areas may be able to provide high levels of coverage of health services and more effective approach to achieve syphilis health education, screening, and optimum treatment services. Therefore, the relevant departments should increase the allocation of health resources to the economically underdeveloped high-risk areas. Additionally, strengthening health education on syphilis and other sexually transmitted diseases, establishing health awareness among people, and promoting conscious adoption of healthy sexual behaviors may minimize viral infections caused by high-risk sexual behavior and may reduce the incidence of syphilis in Ningxia.

The current study has some limitations. First, this study did not provide a detailed sub-population analysis of the different stages of syphilis characteristics due to limited data; thus, we could not provide more precise recommendations for different sub-populations. Second, the data on influencing factors was limited by the data of covariates, such as education, urbanization rate, and population migration and it is recommended that more extensive and detailed research variables be selected for in-depth analysis in subsequent studies. Third, considering the serious health impact of syphilis on adults and its vertical transmission from mother to child, more attention should be paid to strengthening maternal surveillance efforts to minimize congenital syphilis; thus, further studies should address the spatial heterogeneity of congenital syphilis. Finally, this study was an ecological study examining the association between the incidence of syphilis and its influencing socio-economic and health-resource factors, the potential ecological fallacy is inevitable.

## 5. Conclusions

In summary, the results of this study provide empirical evidence for the heterogenous spatiotemporal distribution of syphilis in Ningxia and its influencing socio-economic and health-resource factors. The incidence of syphilis in Ningxia has increased in recent years and there were significantly spatial clusters. Two spatial-temporal clusters were detected, with 2.94- and 2.57-times higher risk of syphilis than that found in other areas. We found that the socio-economic and health-resource factors could affect the incidence of syphilis to different degrees and in different directions. However, sex ratio and meteorological factors were not significantly associated with syphilis across Ningxia. Understanding the spatial heterogeneity of syphilis incidence and associated influencing factors plays an important role in implementing precise and effective public health intervention strategies for the control and prevention of syphilis.

## Figures and Tables

**Table 1:** Results of spatiotemporal scan of syphilis incidence in Ningxia from 2004 to 2017.

Cluster	Time Range	Cluster a Dministrative Area	Center Point (Longitude, Latitude)	Radius (km)	Actual Number of Cases	Expected Number of Cases	*LLR*	*p*	*RR*
1	2011–2017	Huinong, Dawukou, Pingluo, Helan, Xingqing, Jinfeng, Xixia, Yongning, Lingwu, Litong	39.25 N, 106.78 E	149.84	14404	7342.34	3972.88	<0.001	2.94
2	2015–2017	Yuanzhou, Pengyang	36.00 N, 106.28 E	35.63	1482	594.84	479.88	<0.001	2.57

**Table 2:** Global *Moran’s I* of syphilis SMR in Ningxia, 2004–2017.

Year	*Moran* *’s I*	*Z*-Score	*p*-Value	Pattern
2004	0.1625	1.7416	0.0630	Not clustered
2005	0.0685	0.8514	0.1920	Not clustered
2006	0.1412	1.3823	0.0950	Not clustered
2007	0.2369	2.0578	0.0300 *	Clustered
2008	0.1767	1.5987	0.0670	Not clustered
2009	0.3110	2.7119	0.0080 **	Clustered
2010	0.3759	3.0168	0.0050 **	Clustered
2011	0.5161	4.1887	0.0010 ***	Clustered
2012	0.5439	4.2181	0.0020 **	Clustered
2013	0.2642	2.2130	0.0310 *	Clustered
2014	0.1011	1.2528	0.1190	Not clustered
2015	−0.1169	−0.4753	0.3330	Not clustered
2016	−0.0436	0.0271	0.4480	Not clustered
2017	0.0774	0.8742	0.1930	Not clustered

Note: * indicates *p* ≤ 0.05, ** indicates *p* ≤ 0.01, *** indicates *p* ≤ 0.001.

**Table 3:** Descriptive statistics of the study variables.

Variable	N	Mean	Std. Dev.	Min	Max
Syphilis_smr	308.00	0.64	0.64	0.00	3.66
at	308.00	9.42	1.51	5.40	11.70
ht	308.00	27.16	9.81	11.90	41.00
lt	308.00	−9.78	11.94	−26.80	6.20
pr	308.00	275.38	139.83	108.30	919.00
aGDP	308.00	26,609.43	24,660.72	1405.90	149,061.00
h1	308.00	131.24	87.15	5.00	385.00
h2	308.00	1850.83	2074.74	22.00	14,537.00
sex_ratio	308.00	1.05	0.03	0.94	1.25
GDP3	308.00	333,589.63	515,637.41	5023.93	4,288,330.00
GDP3pp	308.00	14,794.01	14,549.95	1773.00	87,307.00

Note: at: average daily temperature, ht: maximum temperature, lt: low temperature, pr: precipitation, aGDP: gross Domestic Product per capita, h1: number of health institutions, h2: number of health facility personnel, sex_ratio: sex ratio = male population/female population, GDP3: the tertiary industry GDP, GDP3pp: number of persons employed in tertiary industry.

**Table 4:** Results of Lagrange multiplier test.

Test		Statistic	df	*p*-Value
Spatial error	*Moran* *’s I*	3.032	1	0.002
	Lagrange multiplier	6.632	1	0.010
	Robust Lagrange multiplier	9.359	1	0.002
Spatial lag	Lagrange multiplier	0.324	1	0.569
	Robust Lagrange multiplier	3.052	1	0.081

**Table 5:** HAUSMAN test.

Syphilis_smr		Coef.	Std. Err.	*z*	*p > z*	95% *CI*	
Main	at	0.115	0.046	2.500	0.012	0.025	0.206
	ht	−0.012	0.016	−0.760	0.449	−0.043	0.019
	lt	−0.028	0.013	−2.210	0.027	−0.053	−0.003
	pr	0.001	0.000	2.590	0.010	0.000	0.001
	aGDP	0.000	0.000	2.280	0.023	0.000	0.000
	h1	−0.001	0.000	−1.690	0.091	−0.002	0.000
	h2	0.000	0.000	0.010	0.996	−0.000	0.000
	sex_ratio	0.063	0.704	0.090	0.928	−1.316	1.443
	LgGDP3	0.358	0.068	5.300	0.000	0.226	0.491
	GDP3pp	−0.000	0.000	−0.530	0.596	−0.000	0.000
	cons	−4.940	1.110	−4.450	0.000	−7.117	−2.764
Spatial	λ	0.533	0.080	6.640	0.000	0.375	0.690
Variance	ln_phi	−0.814	0.440	−1.850	0.064	−1.676	0.049
	$\sigma_{e}^{2}$	0.087	0.008	11.540	0.000	0.072	0.102

Note: at: average daily temperature, ht: maximum temperature, lt: low temperature, pr: precipitation, aGDP: gross Domestic Product per capita, h1: number of health institutions, h2: number of health facility personnel, sex_ratio: sex ratio = male population/female population, GDP3: the tertiary industry GDP, GDP3pp: number of persons employed in tertiary industry. H_0_: difference in coefficients not systematic *χ*^2^ (11) = 24.25; *p* ≥ *χ*^2^ = 0.0117.

**Table 6:** The results of spatial error models with different kinds of fixed effects.

Variables		Individual Fixed Effects	Time Fixed Effects	Two-Way Fixed Effect
Main	ht	0.0298203	0.0324021	0.0243556
		(0.000 ***)	(0.072 *)	(0.349)
	pr	0.0004802	0.0003578	0.000131
		(0.128)	(0.229)	(0.695)
	aGDP	0.00000798	0.00000548	0.00000438
		(0.000 ***)	(0.000 ***)	(0.029 **)
	h1	−0.0000834	−0.0004344	−0.0008761
		(0.871)	(0.324)	(0.094 *)
	h2	−0.0001303	−0.0000988	−0.0001397
		(0.062 *)	(0.034 **)	(0.047 **)
	sex_ratio	0.0215899	0.3500529	0.1528104
		(0.976)	(0.635)	(0.831)
	GDP3	0.000000327	0.000000107	0.000000319
		(0.063 *)	(0.315)	(0.072 *)
	GDP3pp	0.000000886	0.0000204	0.00000112
		(0.940)	(0.000 ***)	(0.927)
Spatial	λ	0.6609336	0.4093769	0.3547828
		(0.000 ***)	(0.000 ***)	(0.000 ***)
Variance	$\sigma_{e}^{2}$	0.0874704	0.1165865	0.0829824
		(0.000 ***)	(0.000 ***)	(0.000 ***)

Note: ht: maximum temperature, pr: precipitation, aGDP: gross Domestic Product per capita, h1: number of health institutions, h2: number of health facility personnel, sex_ratio: sex ratio = male population/female population, GDP3: the tertiary industry GDP, GDP3pp: number of persons employed in tertiary industry. *p*-value in parentheses, * indicates *p* < 0.1, ** indicates *p* < 0.05, *** indicates *p* < 0.01.

**Table 7:** Test analysis for optimal effect selection.

Type	χ^2^-Value	*p*-Value
Comparing individual fixed effects with two-way fixed effects	42.10	<0.0001
Comparing time fixed effects with two-way fixed effects	107.67	<0.0001

**Table 8:** The regression results of spatial error model.

Syphilis_smr		Coef.	Std. Err.	Z	*p* > Z	95% CI	
Main	ht	0.0243556	0.026	0.940	0.349	−0.027	0.075
	pr	0.000131	0.000	0.390	0.695	−0.001	0.001
	**aGDP**	**0.00000438**	**0.000**	**2.180**	**0.029** **	**0.000**	**0.000**
	**h1**	**−0.0008761**	**0.001**	**−1.670**	**0.094** *	**−0.002**	**0.000**
	**h2**	**−0.0001397**	**0.000**	**−1.980**	**0.047** **	**−0.000**	**−0.000**
	sex_ratio	0.1528104	0.715	0.210	0.831	−1.248	1.554
	**GDP3**	**0.000000319**	**0.000**	**1.800**	**0.072** *	**−0.000**	**0.000**
	GDP3pp	0.00000112	0.000	0.090	0.927	−0.000	0.000
Spatial	λ	0.3547828	0.091	3.890	0.000 ***	0.176	0.534
Variance	$\sigma_{e}^{2}$	0.0829824	0.007	12.210	0.000 ***	0.070	0.096

Note: ht: maximum temperature, pr: precipitation, aGDP: gross Domestic Product per capita, h1: number of health institutions, h2: number of health facility personnel, sex_ratio: sex ratio = male population/female population, GDP3: the tertiary industry GDP, GDP3pp: number of persons employed in tertiary industry. *p*-value in parentheses, * indicates *p* < 0.1, ** indicates *p* < 0.05, *** indicates *p* < 0.01. The bold type indicates statistically significant regressors.

## Data Availability

The data used to support the findings of this study are available from the corresponding author upon request.

## Supplementary Materials

The following supporting information can be downloaded at: https://www.mdpi.com/article/10.3390/ijerph191710541/s1, Figure S1: Flow chart of spatial regression model analysis; Figure S2: The incidence of syphilis in Ningxia, 2004–2017; Table S1: List of variable information; Table S2: Spatiotemporal distribution of syphilis SMR in Ningxia, 2004–2017; Table S3: Analysis of local *Moran**’s I* index and spatial aggregation of syphilis SMR in Ningxia from 2004–2017.
