# Supplementary material for: Spatial Heterogeneity and Its Influencing Factors of Syphilis in Ningxia, Northwest China, from 2004 to 2017: A Spatial Analysis
Source: Int J Environ Res Public Health. 2022 Aug 24;19(17):10541. doi: 10.3390/ijerph191710541 (PMC9518519; doi:10.3390/ijerph191710541)
Supplement: Supplementary file 1 [file ijerph-19-10541-s001.zip › ijerph-1777665-supplementary.pdf]

Supplementary material

Table S1. List of variable information

Variable Type	Name	Unit	Meaning	Data Sources
Meteorological factors	ht	℃	Daily maximum temperature	Ningxia Statistical Yearbook (http://nxdata.com.cn/publish.htm?cn=G01)
	lt	℃	Daily low temperature	
	at	℃	Average daily temperature	
	pr	mm	Precipitation	
Socio-economic factors	GDP3	RMB	gross regional domestic product of tertiary industry	
	GDP3pp	person	Number of persons employed in tertiary industry	
Health resources Factors	h1	individual	Number of health institutions	
	h2	person	Number of health facility personnel	

Figure S1. Flow chart of spatial regression model analysis.

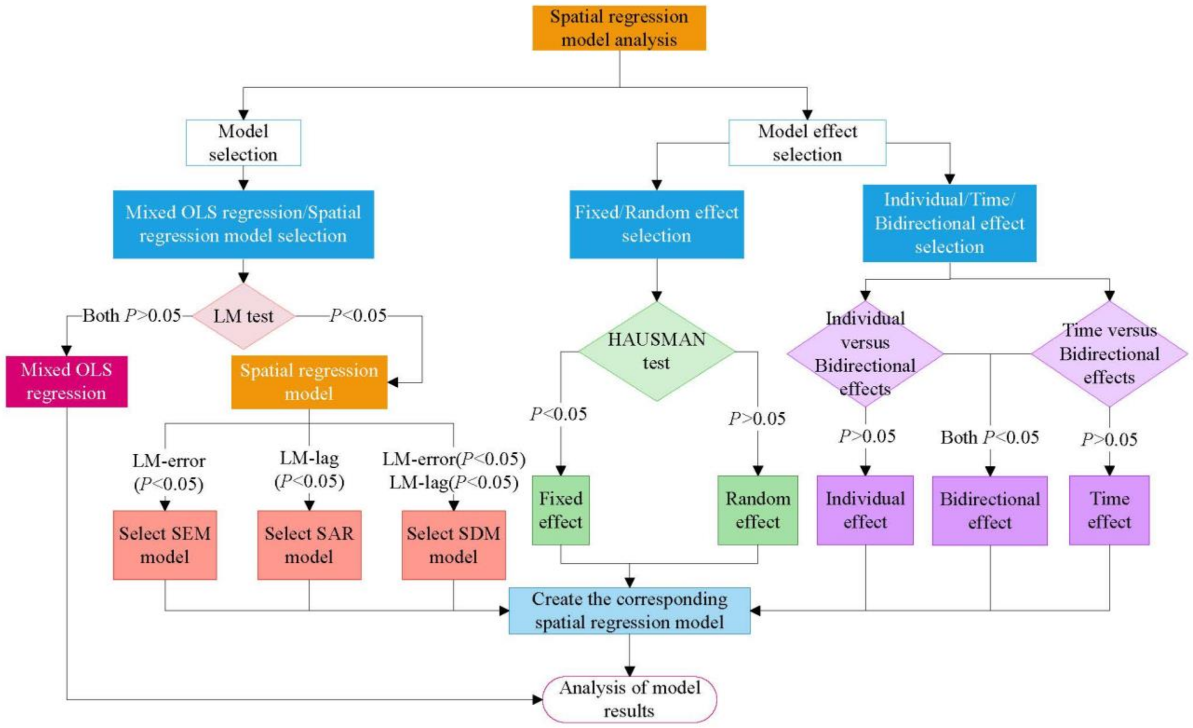

Figure S2. The incidence of syphilis in Ningxia, 2004-2017.

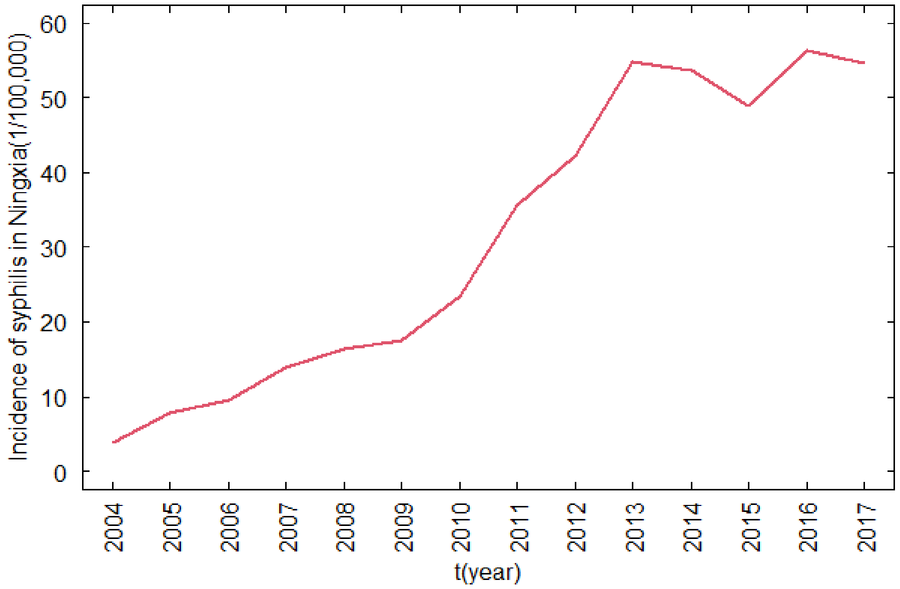

Table S2. Spatiotemporal distribution of syphilis standardized morbidity ratio in Ningxia, 2004-2017.

Year	Min	Max	Mean	Standard deviation	Median	The top 3 administrative areas with the highest SMR values
2004	0.0000	0.2962	0.0463	0.0699	0.0212	Dawukou, Xingqing, Jinfeng
2005	0.0000	0.4566	0.1548	0.1513	0.1062	Yanchi, Litong, Dawukou
2006	0.0000	0.3792	0.1085	0.0992	0.1132	Xingqing, Dawukou, Pingluo
2007	0.0075	0.4568	0.1793	0.1365	0.1844	Xingqing, Jinfeng, Dawukou
2008	0.0123	0.7366	0.2400	0.2106	0.2097	Jinfeng, Xingqing, Xixia
2009	0.0171	0.8534	0.2840	0.2062	0.2922	Xingqing, Xixia, Jinfeng
2010	0.0269	1.7578	0.4516	0.4586	0.3115	Xingqing, Jinfeng, Xixia
2011	0.0934	2.1046	0.6450	0.4792	0.5948	Xingqing, Helan, Xixia
2012	0.1322	2.3480	0.8257	0.5574	0.8998	Xingqing, Helan, Yanchi
2013	0.2051	2.8843	1.1330	0.7408	1.0524	Xingqing, Yanchi, Helan
2014	0.1588	3.6637	1.0345	0.7289	0.9954	Yanchi, Xingqing, Helan
2015	0.2903	2.1116	1.1369	0.4680	1.2218	Yanchi, Xingqing, Helan
2016	0.5772	2.3366	1.3937	0.5787	1.4445	Pengyang, Yongning, Yuanzhou
2017	0.4525	2.2998	1.2626	0.5697	1.2775	Yongning, Helan, Yanchi

Table S3. Analysis of local *Moran's I* index and spatial aggregation of syphilis SMR in Ningxia from 2004-2017.

Year	*Moran's I*	High-High	Low-High	Low-Low	High-Low
2007	0.2369	Jinfeng, Pingluo, Xixia , Xingqing, Helan, Yongning, Lingwu	Huinong	Xiji, Jingyuan, Longde, Pengyang, Hongsibu, Haiyuan, Tongxin, Qingtongxia, Yanchi	Dawukou, Litong, Yuanzhou, Shapotou, Zhongning
2009	0.3110	Jinfeng, Xixia, Xingqing, Helan, Yongning, Lingwu	Pingluo, Huinong	Xiji, Jingyuan, Longde, Pengyang, Hongsibu, Litong, Haiyuan, Tongxin, Zhongning	Dawukou, Yuanzhou, Shapotou, Qingtongxia, Yanchi
2010	0.3759	Jinfeng, Xixia, Xingqing, Huinong, Helan, Yongning	Pingluo, Lingwu	Xijian, Jingyuan, Longde, Pengyang, Hongsibu, Litong, Yuanzhou, Haiyuan, Tongxin, Zhongning, Qingtongxia, Yanchi	Dawukou, Shapotou
2011	0.5161	Jinfeng, Xixia, Xingqing, Huinong, Dawukou, Helan, Yongning, Lingwu	Pingluo	Xiji, Jingyuan, Longde, Pengyang, Hongsibu, Litong, Yuanzhou, Haiyuan, Tongxin, Zhongning, Qingtongxia	Shapotou, Yanchi
2012	0.5439	Jinfeng, Pingluo, Xixia, Xingqing, Huinong, Dawukou, Helan, Yongning, Lingwu	None	Xiji, Jingyuan, Longde, Pengyang, Hongsibu, Yuanzhou, Haiyuan, Tongxin, Zhongning, Qingtongxia	Litong, Shapotou, Yanchi
2013	0.2642	Jinfeng, Xiji, Xixia, Xingqing, Huinong, Helan, Yongning, Litong, Lingwu	Pingluo, Hongsiabao	Xijian, Jingyuan, Longde, Pengyang, Yuanzhou, Haiyuan, Tongxin, Shapotou, Zhongning, Qingtongxia	Dawukou, Yanchi
